# Supplementary material for: Tumor-induced orexigenic imbalance lowers protein appetite and drives early organ wasting symptoms
Source: Nat Commun. 2026 Mar 6;17:3553. doi: 10.1038/s41467-026-70074-2 (PMC13086994; doi:10.1038/s41467-026-70074-2)
Supplement: Supplementary file 1 — Supplementary Information [file 41467_2026_70074_MOESM1_ESM.pdf]

Supplementary Information

Tumor-induced orexigenic imbalance lowers protein appetite and drives early organ wasting symptoms

Afroditi Petsakou[1,2]*, Elizabeth Filine[1], Matthew Li[1], Yuchen Chen[1], Alice Zheng[1], and Norbert Perrimon[1,3]*

[1]*Department of Genetics, Harvard Medical School, Boston, USA*
[2]*Department of Developmental and Molecular Biology, Albert Einstein College of Medicine, Bronx, USA*
[3]*Howard Hughes Medical Institute, Boston, USA*

* Corresponding authors:
Afroditi Petsakou: Afroditi.Petsakou@einsteinmed.edu
Norbert Perrimon: perrimon@genetics.med.harvard.edu

Supplementary Information Index:

Index……………………………………………………………………………………..page 1
Supplementary Figure 1……………………………………..page 2-3
Supplementary Figure 2…………………………………………………………………….page 4
Supplementary Figure 3…………………………………………………………………….page 5-6
Supplementary Figure 4……………………………………..page 7
Supplementary Figure 5…………………………………………………………………….page 8
Genotypes per Figure……………………………………………………………………….page 9-13
Supplementary Table 1……………………………………………………………………..page 14

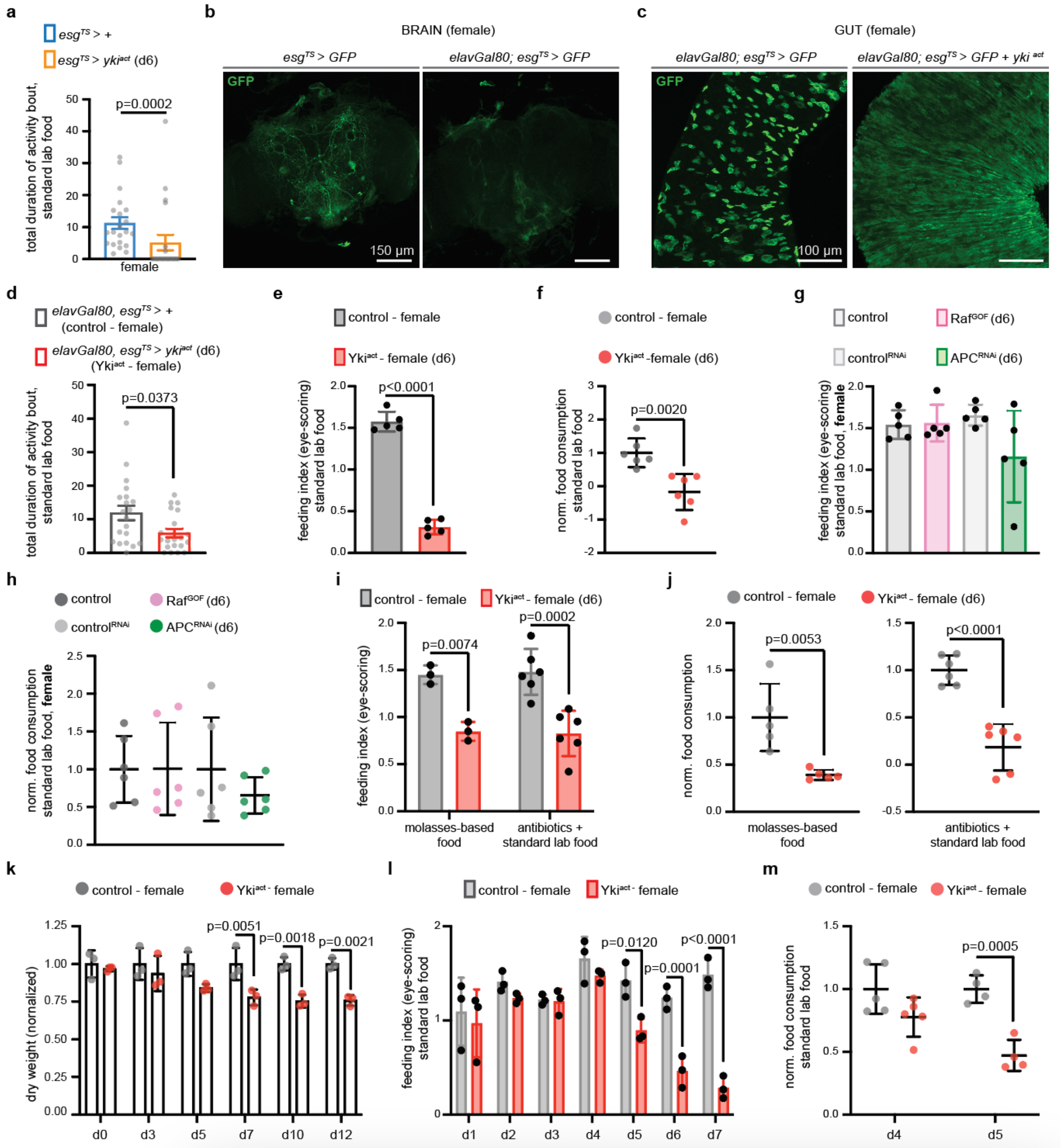

Supplementary Fig. 1: Gut Yki-tumors cause anorexia prior to organ wasting in females.
a. FlyPAD assay. Control (*esg*^{TS}>+, blue) and flies with gut Yki-tumor (*esg*^{TS}>*yki*^{act}, orange) for 6 days. n=21 female flies per genotype, 3 independent experiments. Two-tailed Mann-Whitney test. **b.** Brains of d6 *esg*^{TS}>*GFP* and *elavGal80;esg*^{TS}>*GFP*, 2 independent experiments. **c.** Guts of d6 *elavGal80;esg*^{TS}>*GFP* and *elavGal80;esg*^{TS}>*GFP +yki*^{act}, 2 independent experiments. **d.** FlyPAD assay. Control (*elavGal80;esg*^{TS}>+, grey) and flies with d6 gut-specific Yki-tumor (*elavGal80;esg*^{TS}>*yki*^{act}*,* referred as Yki^{act}, red). n=20 female flies per genotype, 3 independent

experiments. Two-tailed Mann-Whitney test. **e.** Eye-scoring assay. Flies like (d). n=5 biological replicates per genotype. 3 independent experiments. Unpaired two-tailed t-test (t=19.03, df=8). **f.** Blue-dye (spectrophotometry) assay. Flies like (d). Normalized to control. n=6 biological replicates per genotype, 3 independent experiments. Unpaired two-tailed t-test (t=4.150, df=10). **g.** Eye-scoring assay. Control (grey), Raf^{GOF} (*elavGal80;esg*^{TS}*>Raf*^{GOF}, pink), $control^{RNAi}$ (*elavGal80;esg*^{TS}*>Luciferase*^{RNAi}*,* light grey), APC^{RNAi} (*elavGal80;esg*^{TS}*>APC*^{RNAi}, green) d6 females. n=5 biological replicates per genotype, 4 independent experiments. One-way Anova (Tukey's test). **h.** Blue-dye (spectrophotometry) assay. Flies like (g). Raf^{GOF} normalized to control, APC^{RNAi} to $control^{RNAi}$. n=6 biological replicates per genotype, 3 independent experiments. One-way Anova (Tukey's test). **i.** Eye-scoring assay. Genotypes like (d) on d6. Biological replicates per genotype: n=3 (molasses), n=6 (antibiotics), 3 independent experiments. Two-way Anova (Sidak's test). **j.** Blue-dye (spectrophotometry) assay. Genotypes like (i). Normalized to control. Biological replicates per genotype: n=5 (molasses), n=6 (antibiotics), 3 independent experiments. Unpaired two-tailed t-test (molasses: t=3.790, df=8; antibiotics: t=6.882, df=10). **k.** Dry weight assay. Genotypes like (d), d0 to d12. n=3 biological replicates per genotype, timepoint, 3 independent experiments. Normalized to control. Two-way Anova (Sidak test). **l.** Eye-scoring assay. Genotypes like (d), d1-d7. n=3 biological replicates per genotype, timepoint, 3 independent experiments. Two-way Anova (Sidak's test). **m.** Blue-dye (spectrophotometry) assay. Genotypes like (d), d4-d5. Normalized to control per timepoint. Biological replicates per genotype: n=5 (d4); n=4 (d5), 3 independent experiments. Two-way Anova (Sidak's test). Activity bout: seconds. anti-GFP: green. scale bar: 150μm (a), 100μm (b). Mean, ± SEM (a, d), SD (e-m). Exact p-values are shown. Source data are provided as Source Data file.

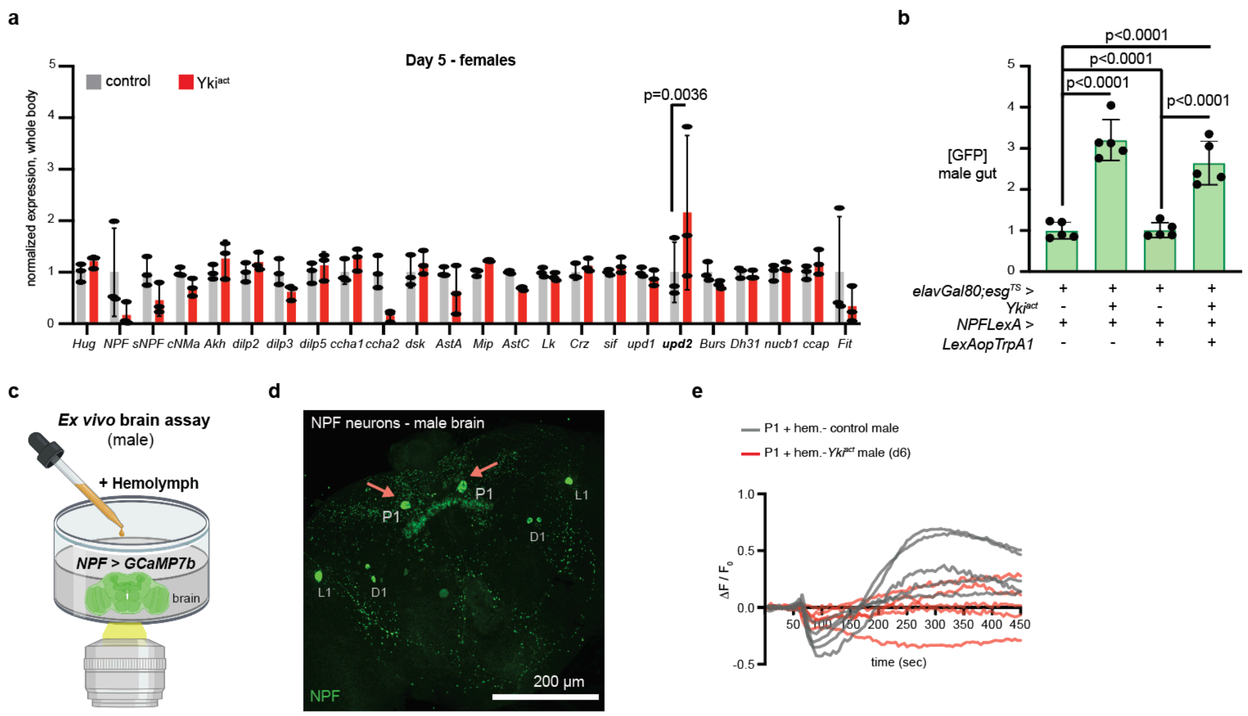

Supplementary Fig. 2: NPF neurons are silenced in Yki^{act} male flies.

a. Expression levels from whole body of female control (*elavGal80; esg^{TS}*>+, grey) and d5 Yki^{act} (*elavGal80;esg^{TS}>yki^{act}*, red). n=3 biological replicates per genotype, 3 independent experiments. Normalized to control. Two-way Anova Sidak's test (additional statistical tests in Supplementary Table 1). **b.** Levels of GFP per posterior gut of male flies as described in Fig. 2e. Normalized to control (*elavGal80;esg^{TS}>GFP* ; *NPFLexA*>+) . n= 5 guts per genotype, 2 independent experiments. One-way Anova (Sidak's test). **c.** Illustration showing *ex vivo* brain live-imaging assay that tests the activity of male NPF neurons after hemolymph addition. Brains from male flies express the Ca^{2+} indicator GCaMP7b in NPF neurons (*NPF>GCAMP7b*). Created in BioRender. Petsakou, A. (2025) https://BioRender.com/wnej2dq. **d.** Confocal image of a brain from male control (*elavGal80; esg^{TS}*>+) stained with anti-NPF (green). red arrows: dorsal median NPF-P1 neurons that were used for imaging. L1: NPF neurons that are part of the clock network. D1: male specific NPF neurons. **e.** Relative fluorescence intensity ($\Delta F/F_0$) per frame (5 seconds per frame) and per genotype of individual male NPF-P1 neurons as described in Fig. 2h. Mean, ± SD. Exact p-values are shown. Source data are provided as Source Data file.

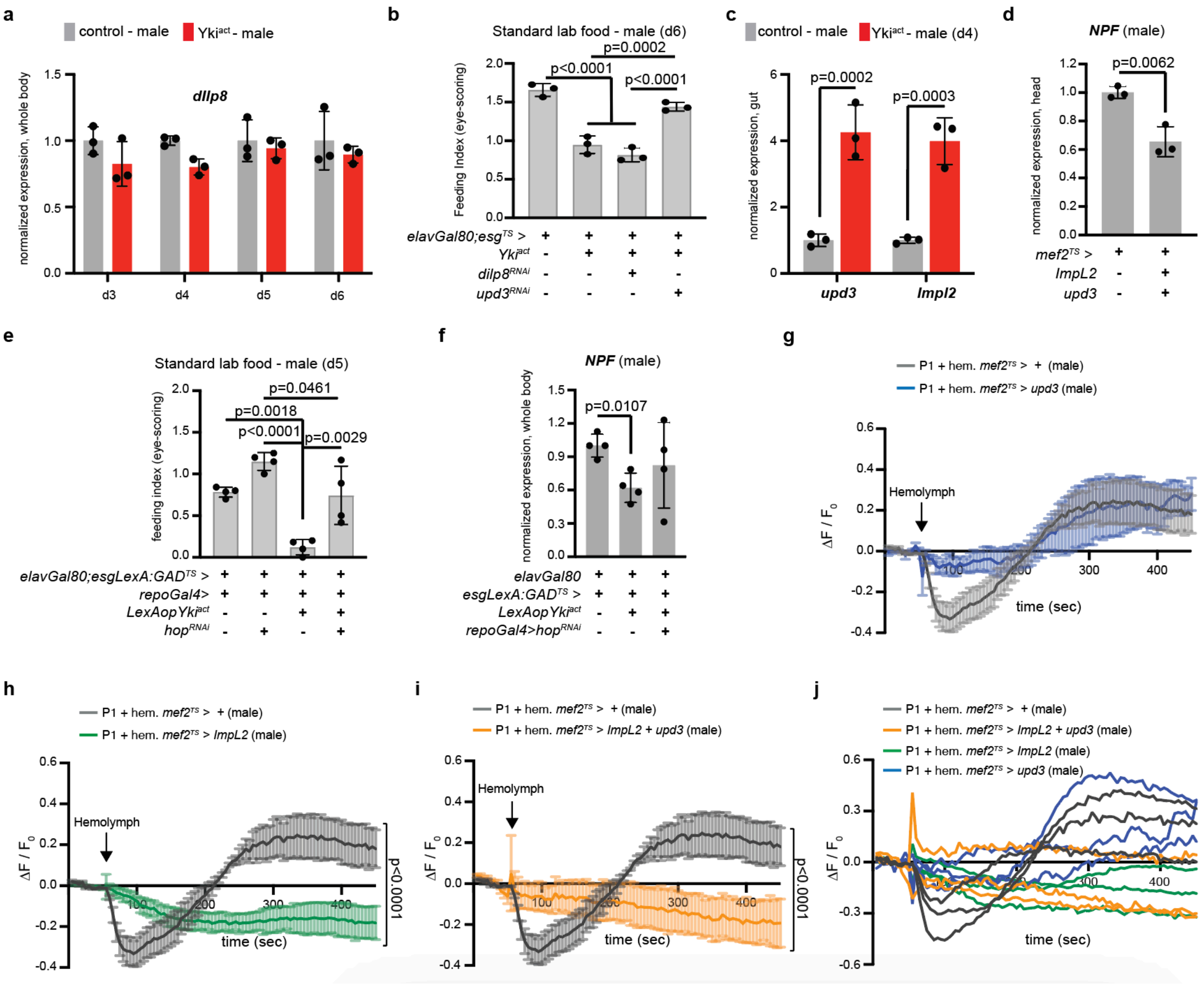

Supplementary Fig. 3: ImpL2 signaling aided by upd3 causes anorexia in male flies. **a.** *dilp8* levels from whole body of male control (grey) and Yki^{act} (red) d3-d6. n=3 biological replicates per genotype, timepoint (3 independent experiments). Normalized to control per timepoint. Two-way Anova (Sidak's test). **b.** Eye-scoring assay on d6. Control, Yki^{act}, *elavGal80;esg^{TS}>yki^{act}+ dilp8^{RNAi}* (Yki^{act} with *dilp8* knockdown in the gut) and *elavGal80;esg^{TS}>yki^{act} + upd3^{RNAi}* (Yki^{act} with *upd3* knockdown in the gut). n= 3 biological replicates per genotype, 3 independent experiments. One-way Anova (Tukey's test). **c.** *upd3* and *ImpL2* levels from gut of control (grey) and Yki^{act} (red) d4 male flies. n=3 biological replicates per genotype. Normalized to control. Two-way Anova (Sidak's test). **d.** *NPF* levels from head of control (*mef2^{TS}>+*) and *mef2^{TS}>ImpL2+upd3* d4 male flies. n=3 biological replicates per genotype, 3 independent experiments. Normalized to control. Unpaired two-tailed t-test (t=5.282, df=4). **e.** Eye-scoring assay on d5. Male control (*elavGal80;esgLexA:GAD^{TS}>+; repoGal4>+*), flies with *hop* knockdown on the BBB (*elavGal80;esgLexA:GAD^{TS}>+; repoGal4>hop^{RNAi}*), flies with gut-specific Yki-tumor (*elavGal80;esgLexA:GAD^{TS}>LexAopYki^{act};*

repoGal4>+), and flies with *hop* knockdown on the BBB alongside gut-specific Yki-tumor (*elavGal80;esgLexA:GAD*^{TS}*>LexAopYki*^{act}; *repoGal4>hop*^{RNAi}). n= 3 biological replicates per genotype, 3 independent experiments. One-way Anova (Tukey's test). **f.** *NPF* levels from whole body of male control (*elavGal80;esgLexA:GAD*^{TS}), flies with gut-specific Yki-tumor (*elavGal80;esgLexA:GAD*^{TS}*>LexAopYki*^{act}) and flies with *hop* knockdown on the BBB alongside gut-specific Yki-tumor (*elavGal80;esgLexA:GAD*^{TS}*>LexAopYki*^{act}; *repoGal4>hop*^{RNAi}) on d5. n=4 biological replicates per genotype, 3 independent experiments. Normalized to control. One-way Anova (Dunnett's T3 test). **g-i.** Relative fluorescence intensity ($\Delta F/F_0$) of NPF-P1 per frame (5 sec/frame) as described in Fig. 3h. P1+ hem. *mef2*^{TS}>+: $\Delta F/F_0$ of P1 with *mef2*^{TS}>+ hemolymph (grey); P1+ hem. *mef2*^{TS}*>upd3*: $\Delta F/F_0$ of P1 with *mef2*^{TS}*>upd3* hemolymph (blue); P1+ hem. *mef2*^{TS}*>ImpL2*: $\Delta F/F_0$ of P1 with *mef2*^{TS}*>ImpL2* hemolymph (green); P1+ hem. *mef2*^{TS}*>ImpL2+upd3*: $\Delta F/F_0$ of P1 with *mef2*^{TS}> *ImpL2+upd3* hemolymph (orange). n=3 neurons per condition and genotype, 3 independent experiments. Two-way Anova (Sidak's test) and two-sided Mann Whittney test. **j.** $\Delta F/F_0$ of individual NPF-P1 as described in (g-i) and in Fig. 3h. Mean $\pm$ SD (a-f), SEM (g-i). Source data are provided as Source Data file.

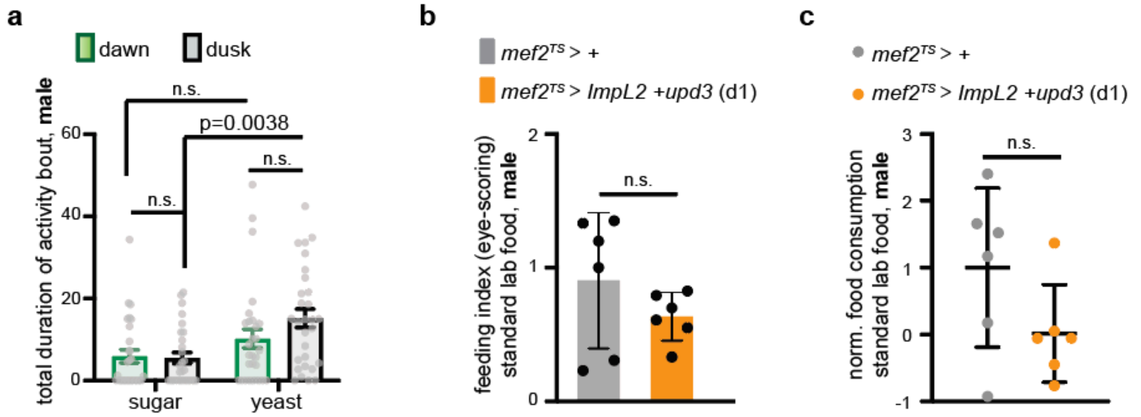

Supplementary Fig. 4: Protein vs sugar food preference is higher at dusk in male flies.

a. FlyPAD two choice-assay of control (*elavGal80;esg*^{TS}>+) male flies at dawn (ZT0-ZT2, green) and dusk (ZT10-ZT12, black) on d4. Activity bout: seconds. n= 29 flies per genotype, 4 independent experiments. Kruskal Wallis Dunn's test. **b-c.** Feeding assays of control (*mef*^{TS}>+) and male flies with *Impl2* and *upd3* (*mef*^{TS}*>ImpL2+upd3,* orange) overexpressed from the muscle for ~18hrs (d1). Eye-scoring assay (b). Blue dye (spectrophotometry) assay (c). n=6 biological replicates per genotype and assay, 3 independent experiments per assay. Unpaired two-tailed t-test (b: t=1.221, df=10; c: t=1.726, df=10). n.s: non-significant. Mean $\pm$ SEM (a), SD (b-c). Exact p-values are shown. Source data are provided as Source Data file.

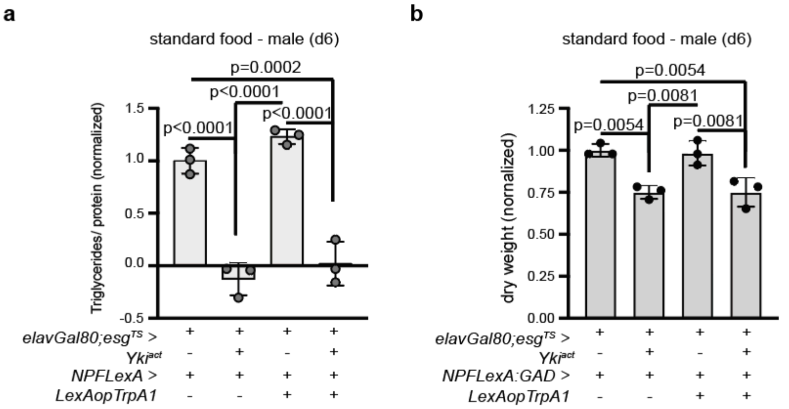

c

**genotypes** (male)	Probability of Survival 75%	**50%**	25%	**0%**
	(days)			
elavGal80;esg^TS > +	30	**37**	39	**46**
NPFLexA > LexAopTrpA1	27	**30**	37	**44**
elavGal80;esg^TS > Yki^act	14	**19**	26	**41**
elavGal80;esg^TS > Yki^act + NPFLexAopTrpA1	17	**24**	30	**46**

Supplementary Fig. 5: NPF increase in Yki^{act} male flies doesn't rescue lipolysis but increases survival.

a. Triglyceride assay. Male control (*elavGal80;esg^{TS}/+;NPFLexA/+*), Yki^{act} (*elavGal80;esg^{TS}>yki^{act}* + *NPFLexA*>+), flies with increased NPF signaling (*NPFLexA>LexAopTrpA1* + *elavGal80;esg^{TS}*>+) and Yki^{act} flies with increased NPF signaling (*NPFLexA>LexAopTrpA1* + *elavGal80;esg^{TS}>yki^{act}*) on d6. Normalized to control. n=3 biological replicates per genotype, 3 independent experiments. One-way Anova (Tukey's test). **b.** Dry weight assay. Male control d6 (*elavGal80;esg^{TS}/+; NPFLexA:GAD/*+), Yki^{act} flies (*elavGal80;esg^{TS}>yki^{act}* + *NPFLexA:GAD*>+), male flies with increased NPF signaling from the gut (*NPFLexA>LexAopTrpA1* + *elavGal80;esg^{TS}*>+) and Yki^{act} flies with increased gut NPF signaling (*NPFLexA>LexAopTrpA1* + *elavGal80;esg^{TS}>yki^{act}*). n=3 biological replicates per genotype, 3 independent experiments. Normalized to control. One-way Anova (Tukey's test). **c**. Table shows the probability of 75%, 50% (median survival), 25% and 0% survival per genotype of male flies as shown in Fig. 5c (the day that the survival curve passes each survival point). Mean ± SD. Exact p-values are shown. Source data are provided as Source Data file.

Genotypes per figure

Figure 1

Esg^{TS}>+ corresponds to *+;esg-Gal4 Tubulin-Gal80*^{TS} *UAS-GFP/UAS-emptyVK37;+*.

Esg^{TS}>*yki*^{act} corresponds to *+;esg-Gal4 Tubulin-Gal80*^{TS} *UAS-GFP/+;UAS- yki*^{S3A}/+.

Esg^{TS}>*GFP* corresponds to *+;esg-Gal4 Tubulin-Gal80*^{TS} *UAS-GFP/+;+*.

ElavGal80;esg^{TS}>*GFP* corresponds to *elav-Gal80;esg-Gal4 Tubulin-Gal80*^{TS} *UAS-GFP/+;+*.

ElavGal80;esg^{TS}>*GFP+ yki*^{act} or Yki^{act} corresponds to *elav-Gal80;esg-Gal4 Tubulin-Gal80*^{TS} *UAS-GFP/+;UAS-yki*^{S3A}/+.

Control or *elavGal80;esg*^{TS}>+ corresponds to *elav-Gal80;esg-Gal4 Tubulin-Gal80*^{TS} *UAS-GFP/UAS-emptyVK37;+*.

Raf^{GOF} corresponds to *elav-Gal80;esg-Gal4 Tubulin-Gal80*^{TS} *UAS-GFP/+;UAS-RAF*^{179}/+.

Control^{RNAi} corresponds to *elav-Gal80;esg-Gal4 Tubulin-Gal80*^{TS} *UAS-GFP/+;UAS-Luciferase*^{RNAi}/+.

APC^{RNAi} corresponds to *elav-Gal80;esg-Gal4 Tubulin-Gal80*^{TS} *UAS-GFP/+;UAS-APC*^{RNAi}/+.

Figure 2

Control corresponds to *elav-Gal80;esg-Gal4 Tubulin-Gal80*^{TS} *UAS-GFP/UAS-emptyVK37;+*.

Yki^{act} corresponds to *elav-Gal80;esg-Gal4 Tubulin-Gal80*^{TS} *UAS-GFP/+;UAS- yki*^{S3A}/+.

ElavGal80; esg^{TS}>*+;NPFLexA*>+ corresponds to *elav-Gal80;esg-Gal4 Tubulin-Gal80*^{TS} *UAS-GFP/+;NPF-LexA/+*.

ElavGal80;esg^{TS}>*yki*^{act}*; NPFLexA*>+ corresponds to *elav-Gal80;esg-Gal4 Tubulin-Gal80*^{TS} *UAS-GFP /+;NPF-LexA/UAS-yki*^{S3A}.

ElavGal80;esg^{TS}>*+;NPFLexA>LexAopTrpA1* corresponds to *elav-Gal80;esg-Gal4 Tubulin-Gal80*^{TS} *UAS-GFP/13x-LexAop2-IVS-TrpA1;NPF-LexA/ +*.

ElavGal80; esg^{TS}>*yki*^{act}*; NPFLexA>LexAopTrpA1* corresponds to *elav-Gal80;esg-Gal4 Tubulin-Gal80*^{TS} *UAS-GFP/13x-LexAop2-IVS-TrpA1;NPF-LexA/UAS-yki*^{S3A}.

ElavGal80;esg^{TS}>*GFP; NPFLexA*>+ corresponds to *elav-Gal80;esg-Gal4 Tubulin-Gal80*^{TS} *UAS-GFP/UAS-emptyVK37; NPF-LexA/+*.

ElavGal80; esg^{TS}>*GFP* + Yki^{act} *; NPFLexA*>+ corresponds to *elav-Gal80;esg-Gal4 Tubulin-Gal80*^{TS} *UAS-GFP / +; NPF-LexA/UAS-yki*^{S3A}.

ElavGal80; esg^{TS}>*GFP + NPFLexA>LexAopTrpA1* corresponds to *elav-Gal80;esg-Gal4 Tubulin-Gal80*^{TS} *UAS-GFP/13x-LexAop2-IVS-TrpA1; NPF-LexA/+*.

ElavGal80; esg^{TS}>*GFP + NPFLexA>LexAopTrpA1+* Yki^{act} corresponds to *elav-Gal80; esg-Gal4 Tubulin-Gal80*^{TS} *UAS-GFP/13x-LexAop2-IVS-TrpA1;NPF-LexA/UAS-yki*^{S3A}.

ElavGal80; esg^{TS}>+;NPFLexA:GAD>+ corresponds to *elav-Gal80;esg-Gal4 Tubulin-Gal80^{TS} UAS-GFP/+; NPF-LexA:GAD/+*.
ElavGal80; esg^{TS}>yki^{act}; NPFLexA:GAD>+ corresponds to *elav-Gal80;esg-Gal4 Tubulin-Gal80^{TS} UAS-GFP/+; NPF-LexA:GAD/UAS-yki^{S3A}*.
ElavGal80; esg^{TS}>+ ; NPFLexA:GAD>LexAopTrpA1 corresponds to *elav-Gal80; esg-Gal4 Tubulin-Gal80^{TS} UAS-GFP/13x-LexAop2-IVS-TrpA1; NPF-LexA:GAD/+*.
ElavGal80; esg^{TS}>yki^{act} ; NPFLexA:GAD>LexAopTrpA1 corresponds to *elav-Gal80; esg-Gal4 Tubulin-Gal80^{TS} UAS-GFP/13x-LexAop2-IVS-TrpA1;NPF-LexA:GAD/UAS-yki^{S3A}*.
NPF>GCAM7b corresponds to +*; NPF-Gal4/ 20XUAS-6XmCherry-HA;20XUAS-IVS-jGCaMP7b/+*.

Figure 3

Control corresponds to *elav-Gal80;esg-Gal4 Tubulin-Gal80^{TS} UAS-GFP/UAS-emptyVK37;+*.
Yki^{act} corresponds to *elav-Gal80;esg-Gal4 Tubulin-Gal80^{TS} UAS-GFP/+; UAS- yki^{S3A}/+*.
ElavGal80;esg^{TS}>+ corresponds to *elav-Gal80;esg-Gal4 Tubulin-Gal80^{TS} UAS-GFP/UAS-emptyVK37;+*.
ElavGal80;esg^{TS}>ImpL2^{RNAi} corresponds to *elav-Gal80;esg-Gal4 Tubulin-Gal80^{TS} UAS-GFP /UAS-ImpL2^{RNAi};+/+*.
ElavGal80;esg^{TS}>yki^{act} corresponds to *elav-Gal80;esg-Gal4 Tubulin-Gal80^{TS} UAS-GFP /+;+ /UAS-yki^{S3A}.*
ElavGal80;esg^{TS}>ImpL2^{RNAi} + yki^{act} corresponds to *elav-Gal80;esg-Gal4 Tubulin-Gal80^{TS} UAS-GFP /UAS-ImpL2^{RNAi};+/ UAS-yki^{S3A}*.
Mef2^{TS}>+ corresponds to +*; Tubulin-Gal80^{TS}/UAS-emptyVK37;mef2-Gal4/+*.
Mef2^{TS}>Impl2 corresponds to +*;Tubulin-Gal80^{TS}/UAS-emptyVK37;mef2-Gal4/UAS-ImpL2*.
Mef2^{TS}>upd3 corresponds to +*;Tubulin-Gal80^{TS}/UAS-upd3 ; mef2-Gal4/UAS-nuclear GFP*.
Mef2^{TS}>upd3+Impl2 corresponds to +*;Tubulin-Gal80^{TS}/UAS-upd3;mef2-Gal4/UAS-ImpL2*.
NPF>GCAM7b corresponds to +*; NPF-Gal4/ 20XUAS-6XmCherry-HA;20XUAS-IVS-jGCaMP7b/+*.

Figure 4

Control corresponds to *elav-Gal80;esg-Gal4 Tubulin-Gal80^{TS} UAS-GFP/UAS-emptyVK37;+*.
Yki^{act} corresponds to *elav-Gal80;esg-Gal4 Tubulin-Gal80^{TS} UAS-GFP/+; UAS- yki^{S3A}/+*.
Mef2^{TS}>+ corresponds to +*; Tubulin-Gal80^{TS}/UAS-emptyVK37;mef2-Gal4/+*.
Mef2^{TS}>upd3+Impl2 corresponds to +*;Tubulin-Gal80^{TS}/UAS-upd3;mef2-Gal4/UAS-ImpL2*.

ElavGal80; esg^{TS}>+ corresponds to *elav-Gal80;esg-Gal4 Tubulin-Gal80*^{TS} *UAS-GFP/+;+/+*.
ElavGal80;esg^{TS}>*yki*^{act} corresponds to *elav-Gal80;esg-Gal4 Tubulin-Gal80*^{TS} *UAS-GFP/+;+/UAS-yki*^{S3A}.
NPFLexA>LexAopTrpA1 corresponds to *13x-LexAop2-IVS-TrpA1/+;NPF-LexA/* +.
ElavGal80; esg^{TS}>*yki*^{act}*; NPFLexA>LexAopTrpA1* corresponds to *elav-Gal80;esg-Gal4 Tubulin-Gal80*^{TS} *UAS-GFP/13x-LexAop2-IVS-TrpA1;NPF-LexA/UAS-yki*^{S3A}.
NPFLexA:GAD>LexAopTrpA1 corresponds to *13x-LexAop2-IVS-TrpA1/+;NPF-LexA:GAD/*+.
ElavGal80; esg^{TS}>*yki*^{act} *; NPFLexA:GAD>LexAopTrpA1* corresponds to *elav-Gal80; esg-Gal4 Tubulin-Gal80*^{TS} *UAS-GFP/13x-LexAop2-IVS-TrpA1;NPF-LexA:GAD/UAS-yki*^{S3A}.

Figure 5
ElavGal80; esg^{TS}>+*;NPFLexA*>+ corresponds to *elav-Gal80;esg-Gal4 Tubulin-Gal80*^{TS} *UAS-GFP/+;NPF-LexA/*+.
ElavGal80;esg^{TS}>*yki*^{act}*; NPFLexA*>+ corresponds to *elav-Gal80;esg-Gal4 Tubulin-Gal80*^{TS} *UAS-GFP /+;NPF-LexA/UAS-yki*^{S3A}.
ElavGal80;esg^{TS}>+*;NPFLexA>LexAopTrpA1* corresponds to *elav-Gal80;esg-Gal4 Tubulin-Gal80*^{TS} *UAS-GFP/13x-LexAop2-IVS-TrpA1;NPF-LexA/*+.
ElavGal80; esg^{TS}>*yki*^{act}*; NPFLexA>LexAopTrpA1* corresponds to *elav-Gal80;esg-Gal4 Tubulin-Gal80*^{TS} *UAS-GFP/13x-LexAop2-IVS-TrpA1;NPF-LexA/UAS-yki*^{S3A}.
Mef2^{TS}>+ corresponds to +*; Tubulin-Gal80*^{TS}*/UAS-emptyVK37;mef2-Gal4/*+.
Mef2^{TS}>*Impl2* corresponds to +*;Tubulin-Gal80*^{TS}*/UAS-emptyVK37;mef2-Gal4/UAS-ImpL2*.
Mef2^{TS}>*upd3* corresponds to +*;Tubulin-Gal80*^{TS}*/UAS-upd3 ; mef2-Gal4/UAS-nuclear GFP*.
Mef2^{TS}>*upd3+Impl2* corresponds to +*;Tubulin-Gal80*^{TS}*/UAS-upd3;mef2-Gal4/UAS-ImpL2*.

Supplementary Fig.1
Esg^{TS}>+ corresponds to +*;esg-Gal4 Tubulin-Gal80*^{TS} *UAS-GFP/UAS-emptyVK37;*+.
Esg^{TS}>*yki*^{act} corresponds to +*;esg-Gal4 Tubulin-Gal80*^{TS} *UAS-GFP/+;UAS- yki*^{S3A}*/*+.
Esg^{TS}>*GFP* corresponds to +*;esg-Gal4 Tubulin-Gal80*^{TS} *UAS-GFP/+;*+.
ElavGal80;esg^{TS}>*GFP* corresponds to *elav-Gal80;esg-Gal4 Tubulin-Gal80*^{TS} *UAS-GFP/+;*+.
ElavGal80;esg^{TS}>*GFP+ yki*^{act} or Yki^{act} corresponds to *elav-Gal80;esg-Gal4 Tubulin-Gal80*^{TS} *UAS-GFP/+;UAS-yki*^{S3A}*/*+.
Control or *elavGal80;esg*^{TS}>+ corresponds to *elav-Gal80;esg-Gal4 Tubulin-Gal80*^{TS} *UAS-GFP/UAS-emptyVK37;*+.
Raf^{GOF} corresponds to *elav-Gal80;esg-Gal4 Tubulin-Gal80*^{TS} *UAS-GFP/+;UAS-RAF*^{179}*/*+.

Control^{RNAi} corresponds to *elav-Gal80;esg-Gal4 Tubulin-Gal80*^{TS} *UAS-GFP/+;UAS-Luciferase*^{RNAi}/+.
APC^{RNAi} corresponds to *elav-Gal80;esg-Gal4 Tubulin-Gal80*^{TS} *UAS-GFP/+;UAS-APC*^{RNAi}/+.

Supplementary Fig. 2
Control or *elavGal80;esg*^{TS}>+ corresponds to *elav-Gal80; esg-Gal4 Tubulin-Gal80*^{TS} *UAS-GFP/UAS-emptyVK37;+*.
Yki^{act} corresponds to *elav-Gal80;esg-Gal4 Tubulin-Gal80*^{TS} *UAS-GFP/+;UAS- yki*^{S3A}/+.
ElavGal80; esg^{TS}*/+;NPFLexA/+* corresponds to *elav-Gal80;esg-Gal4 Tubulin-Gal80*^{TS} *UAS-GFP/+;NPF-LexA/+*.
ElavGal80;esg^{TS}*>yki*^{act}*; NPFLexA/+* corresponds to *elav-Gal80;esg-Gal4 Tubulin-Gal80*^{TS} *UAS-GFP /+;NPF-LexA/UAS-yki*^{S3A}.
ElavGal80;esg^{TS}*>+;NPFLexA>LexAopTrpA1* corresponds to *elav-Gal80;esg-Gal4 Tubulin-Gal80*^{TS} *UAS-GFP/13x-LexAop2-IVS-TrpA1;NPF-LexA/+*.
ElavGal80; esg^{TS}*>yki*^{act}*; NPFLexA>LexAopTrpA1* corresponds to *elav-Gal80;esg-Gal4 Tubulin-Gal80*^{TS} *UAS-GFP/13x-LexAop2-IVS-TrpA1;NPF-LexA/UAS-yki*^{S3A}.
NPF> GCAM7b and P1 correspond to +*; NPF-Gal4/ 20XUAS-6XmCherry-HA; 20XUAS-IVS-jGCaMP7b/+*.

Supplementary Fig. 3
Control corresponds to *elav-Gal80;esg-Gal4 Tubulin-Gal80*^{TS} *UAS-GFP/UAS-emptyVK37;+*.
Yki^{act} corresponds to *elav-Gal80;esg-Gal4 Tubulin-Gal80*^{TS} *UAS-GFP/+; UAS- yki*^{S3A}/+.
ElavGal80;esg^{TS}>+ corresponds to *elav-Gal80;esg-Gal4 Tubulin-Gal80*^{TS} *UAS-GFP/UAS-emptyVK37;+*.
ElavGal80;esg^{TS}*>yki*^{act} corresponds to *elav-Gal80;esg-Gal4 Tubulin-Gal80*^{TS} *UAS-GFP /+;+ /UAS-yki*^{S3A}*.*
ElavGal80;esg^{TS}*>dilp8*^{RNAi} + *yki*^{act} corresponds to *elav-Gal80;esg-Gal4 Tubulin-Gal80*^{TS} *UAS-GFP /UAS-dilp8*^{RNAi}*;+/ UAS-yki*^{S3A}*.*
ElavGal80;esg^{TS}*>upd3*^{RNAi} *+yki*^{act} corresponds to *elav-Gal80;esg-Gal4 Tubulin-Gal80*^{TS} *UAS-GFP /+;UAS-upd3-RNAi/ UAS-yki*^{S3A}.
Mef2^{TS}>+ corresponds to +*; Tubulin-Gal80*^{TS}*/UAS-emptyVK37;mef2-Gal4/+*.
Mef2^{TS}*>Impl2* corresponds to +*;Tubulin-Gal80*^{TS}*/UAS-emptyVK37;mef2-Gal4/UAS-ImpL2*.
Mef2^{TS}*>upd3* corresponds to +*;Tubulin-Gal80*^{TS}*/UAS-upd3 ; mef2-Gal4/UAS-nuclear GFP*.
Mef2^{TS}*>upd3+Impl2* corresponds to +*;Tubulin-Gal80*^{TS}*/UAS-upd3;mef2-Gal4/UAS-ImpL2*.

ElavGal80;esgLexA:GAD^{TS}*> +; repoGal4>+* corresponds to *elav-Gal80;esgLexA:GAD, Tubulin-Gal80*^{TS}*/ +; repo-Gal4/+*.
ElavGal80;esgLexA:GAD^{TS}*> +; repoGal4>+* corresponds to *elav-Gal80;esg-LexA:GAD, Tubulin-Gal80*^{TS}*/ +; repo-Gal4/+*.
ElavGal80;esgLexA:GAD^{TS}*> +; repoGal4>hop*^{RNAi} corresponds to *elav-Gal80;esg-LexA:GAD, Tubulin-Gal80*^{TS}*/ +; repo-Gal4/UAS-hop*^{RNAi}.
ElavGal80;esgLexA:GAD^{TS}*> LexAopYki*^{act}*; repoGal4>+* corresponds to *elav-Gal80;esg-LexA:GAD, Tubulin-Gal80*^{TS}*/ LexAop-Yki3SA-GFP; repo-Gal4/+*.
ElavGal80;esgLexA:GAD^{TS}*> LexAopYki*^{act}*; repoGal4>hop*^{RNAi} corresponds to *elav-Gal80;esg-LexA:GAD, Tubulin-Gal80*^{TS}*/ LexAop-Yki3SA-GFP; repo-Gal4/ UAS-hop*^{RNAi}.
P1 corresponds to +*; NPF-Gal4/ 20XUAS-6XmCherry-HA; 20XUAS-IVS-jGCaMP7b/+*.

<u>Supplementary Fig. 4</u>
Control corresponds to *elav-Gal80;esg-Gal4 Tubulin-Gal80*^{TS} *UAS-GFP/UAS-emptyVK37;+*.
Mef2^{TS}*>upd3+Impl2* corresponds to *+;Tubulin-Gal80*^{TS}*/UAS-upd3;mef2-Gal4/UAS-ImpL2*.

<u>Supplementary Fig. 5</u>
ElavGal80; esg^{TS}*>+;NPFLexA>+* corresponds to *elav-Gal80;esg-Gal4 Tubulin-Gal80*^{TS} *UAS-GFP/+;NPF-LexA/+*.
ElavGal80;esg^{TS}*>yki*^{act}*; NPFLexA>+* corresponds to *elav-Gal80;esg-Gal4 Tubulin-Gal80*^{TS} *UAS-GFP /+;NPF-LexA/UAS-yki*^{S3A}.
ElavGal80;esg^{TS}*>+;NPFLexA>LexAopTrpA1* corresponds to *elav-Gal80;esg-Gal4 Tubulin-Gal80*^{TS} *UAS-GFP/13x-LexAop2-IVS-TrpA1;NPF-LexA/+*.
ElavGal80; esg^{TS}*>yki*^{act}*; NPFLexA>LexAopTrpA1* corresponds to *elav-Gal80;esg-Gal4 Tubulin-Gal80*^{TS} *UAS-GFP/13x-LexAop2-IVS-TrpA1;NPF-LexA/UAS-yki*^{S3A}.
ElavGal80; esg^{TS}*>+;NPFLexA:GAD>+* corresponds to *elav-Gal80;esg-Gal4 Tubulin-Gal80*^{TS} *UAS-GFP/+; NPF-LexA:GAD/+*.
ElavGal80; esg^{TS}*>yki*^{act}*; NPFLexA:GAD>+* corresponds to *elav-Gal80;esg-Gal4 Tubulin-Gal80*^{TS} *UAS-GFP/+; NPF-LexA:GAD/UAS-yki*^{S3A}.
ElavGal80; esg^{TS}*>+ ; NPF-LexA:GAD>LexAopTrpA1* corresponds to *elav-Gal80; esg-Gal4 Tubulin-Gal80*^{TS} *UAS-GFP/13x-LexAop2-IVS-TrpA1; NPF-LexA:GAD/+*.
ElavGal80; esg^{TS}*>yki*^{act} *; NPFLexA:GAD>LexAopTrpA1* corresponds to *elav-Gal80; esg-Gal4 Tubulin-Gal80*^{TS} *UAS-GFP/13x-LexAop2-IVS-TrpA1;NPF-LexA:GAD/UAS-yki*^{S3A}.

Supplementary Table 1

Supplementary Table 1 depicts comparisons of feeding related genes between Yki^{act} and control (d5) flies using different two-way Anova multiple comparisons test with separate comparisons for male and female as described in Figure 2a (males) and Supplementary Figure 2a (females). Sidak's test corrects for multiple comparisons and is less likely to include false positives which is why in Figure 2a and Supplementary Figure 2a. we show comparisons with Sidak's test. Exact p-values are shown, ns: non-significant. Supplementary Table 1 is included in the Source Data file.

Two-way Anova multiple comparisons test	**Sidak's**		**Uncorrected Fisher's LSD**	
feeding related genes	**Male**	**Female**	**Male**	**Female**
hugin	ns	ns	ns	ns
NPF	p=0.0454	ns	p=0.0019	p=0.0055
sNPF	ns	ns	ns	ns
CNMa	ns	ns	ns	ns
AKH	ns	ns	p=0.0310	ns
dilp2	ns	ns	ns	ns
dilp3	ns	ns	p=0.0213	ns
dilp5	ns	ns	p=0.0495	ns
ccha1	ns	ns	ns	ns
ccha2	p<0.0001	ns	p<0.0001	p=0.0050
dsk	ns	ns	ns	ns
AstA	ns	ns	ns	ns
MIp	ns	ns	ns	ns
AstC	ns	ns	p=0.0413	ns
LK	ns	ns	ns	ns
CRZ	ns	ns	ns	ns
SIF	ns	ns	ns	ns
upd1	ns	ns	ns	ns
upd2	ns	p=0.0036	ns	p=0.0001
burs	ns	ns	p=0.0062	ns
Dh31	ns	ns	ns	ns
Nucb1	ns	ns	ns	ns
ccap	ns	ns	p=0.0157	ns
FIT	ns	ns	ns	p=0.0268
