# Supplementary material for: Tumor-induced orexigenic imbalance lowers protein appetite and drives early organ wasting symptoms
Source: Nat Commun. 2026 Mar 6;17:3553. doi: 10.1038/s41467-026-70074-2 (PMC13086994; doi:10.1038/s41467-026-70074-2)
Supplement: Supplementary file 2 — Description of Additional Supplementary Files [file 41467_2026_70074_MOESM2_ESM.pdf]

Description of Additional Supplementary Files:

Supplementary Data 1: Primer sequences used for qPCR

Supplementary Movie 1: P1 neuron with control hemolymph (*elavGal80;esg*^{TS}>+ hemolymph).

Supplementary Movie 2: P1 neuron with Ykiact hemolymph (*elavGal80;esg*^{TS}>Ykiact hemolymph).

Supplementary Movie 3: P1 neuron with control-2 hemolymph (*mef2*^{TS}>+ hemolymph)

Supplementary Movie 4: P1 neuron with upd3 hemolymph (*mef2*^{TS}>*upd3* hemolymph)

Supplementary Movie 5: P1 neuron with ImpL2 hemolymph (*mef2*^{TS}>*ImpL2* hemolymph)

Supplementary Movie 6: P1 neuron with ImpL2 + upd3 hemolymph (*mef2*^{TS}>*ImpL2+upd3* hemolymph)
